# Breast MRI increases the number of mastectomies for ductal cancers, but decreases them for lobular cancers

**DOI:** 10.1007/s10549-017-4117-8

**Published:** 2017-01-28

**Authors:** Marc B.I. Lobbes, Ingeborg J.H. Vriens, Annelotte C.M. van Bommel, Grard A.P. Nieuwenhuijzen, Marjolein L. Smidt, Liesbeth J. Boersma, Thijs van Dalen, Carolien Smorenburg, Henk Struikmans, Sabine Siesling, Adri C. Voogd, Vivianne C.G. Tjan-Heijnen

**Affiliations:** 1 Maastricht University Medical Center, Department of Radiology and Nuclear Medicine, 5800, 6202 AZ Maastricht, The Netherlands; 2 Maastricht University Medical Center, Department of Medical Oncology, Maastricht, The Netherlands; 3 Leiden University Medical Center, Leiden, The Netherlands; 4 Catharina Hospital, Department of Surgery, Eindhoven, The Netherlands; 5 Maastricht University Medical Center, Department of Surgery, Maastricht, The Netherlands; 6 Maastricht University Medical Center, Department of Radiation Oncology (MAASTRO Clinic), Maastricht, The Netherlands; 7 Diakonessenhuis Utrecht, Utrecht, The Netherlands; 8 Antoni van Leeuwenhoek Hospital, Amsterdam, The Netherlands; 9 Medical Center Haaglanden, The Hague, The Netherlands; 10 Comprehensive Cancer Organisation the Netherlands, Utrecht, The Netherlands; 11 MIRA Institute of Biomedical Technology and Technical Medicine, Department of Health Technology and Services Research, University of Twente, Enschede, The Netherlands; 12 Maastricht University, Department of Epidemiology, Maastricht, The Netherlands; 13 GROW School for Oncology and Developmental Biology, Maastricht, The Netherlands

**Keywords:** Breast cancer, MRI, Lobular cancer, Surgery, Contralateral breast cancer

## Abstract

**Purpose:**

In this retrospective population-based cohort study, we analyzed breast MRI use and its impact on type of surgery, surgical margin involvement, and the diagnosis of contralateral breast cancer.

**Methods:**

All Dutch patients with cT_1–4_N_0–3_M_0_ breast cancer diagnosed in 2011–2013 and treated with primary surgery were eligible for inclusion. Using multivariable analyses, we analyzed in different categories whether MRI use was related to surgery type, margin involvement, and diagnosis of contralateral breast cancer (CBC).

**Results:**

MRI was performed in 10,740 out of 36,050 patients (29.8%). Patients with invasive ductal cancer undergoing MRI were more likely to undergo primary mastectomy than those without MRI (OR 1.30, 95% confidence interval (CI) 1.22–1.39, *p* < 0.0001). Patients with invasive lobular cancer undergoing MRI were less likely to undergo primary mastectomy than those without MRI (OR 0.86, 95% CI 0.76–0.99, *p* = 0.0303). A significantly lower risk of positive surgical margins after breast-conserving surgery was only seen in patients with lobular cancer who had undergone MRI as compared to those without MRI (OR 0.59, 95% CI 0.44–0.79, *p* = 0.0003) and, consequently, a lower risk of secondary mastectomy (OR 0.61, 95% CI 0.42–0.88, *p* = 0.0088). Patients who underwent MRI were almost four times more likely to be diagnosed with CBC (OR 3.55, 95% CI 3.01–4.17, *p* < 0.0001).

**Conclusions:**

Breast MRI use was associated with a reduced number of mastectomies and less positive surgical margins in invasive lobular cancer, but with an increased number of mastectomies in ductal cancers. Breast MRI use was associated with a fourfold higher incidence of CBC.

## Introduction

In the Netherlands, approximately 14,000 patients are diagnosed with invasive breast cancer annually. To establish an adequate treatment plan for these patients, conventional imaging (i.e., mammography and ultrasound) and tissue sampling are usually performed, with breast MRI being frequently used as additional imaging tool.

Breast MRI is the most accurate imaging modality to evaluate tumor diameter, multifocality, and presence of contralateral breast cancer [1]. Although its sensitivity is 90%, its specificity is 75% [2], frequently causing false-positive findings requiring additional (follow-up) exams, biopsies, or even mastectomies.

According to the 2012 Dutch Breast Cancer guidelines [3, 4], the use of breast MRI can be considered preoperatively in invasive breast cancers for the following indications: (1) when the aim is to perform BCT and a tumor size discrepancy is observed between physical examination, mammography and/or ultrasound, or (2) in patients with ILC (unless the tumor is unifocal in a highly reliable mammogram), especially when the patients are young women. Breast MRI use remains a topic of debate, since meta-analyses have shown that preoperative evaluation using breast MRI might lead to higher mastectomy rates without improvements in re-excision rates after BCT [5, 6]. Consequently, a large variation in the use of preoperative breast MRI in breast cancer patients exists in the Netherlands [7].

In this study, we aimed to analyze the extent and determinants of breast MRI use in patients with invasive breast cancer and its impact on primary surgical treatment, surgical resection margins, and the diagnosis of contralateral breast cancer.

## Methods

This study included all patients treated with surgery for primary invasive breast cancer in the Netherlands in the period of 2011–2013, who had no distant metastases at the time of diagnosis (i.e., cT_1−4_N_0−3_M_0_). Patients not surgically treated, patients with DCIS, patients undergoing neoadjuvant systemic therapy, and patients with unknown tumor localization within the breast were excluded from final analysis (*n* = 17,859). Due to its retrospective design, the current study does not, under Dutch law, require medical ethics approval.

In daily Dutch practice, breast cancer patients initially undergo mammography and/or ultrasound in combination with tissue sampling of culprit lesions. Axillary ultrasound is performed as axillary staging modality. Next, these patients are discussed in multidisciplinary tumor board meetings, in which the necessity to perform additional breast MRI is discussed while considering our national guidelines. Breast MRI protocols in the Netherlands all adhere to the quality criteria suggested by EUSOBI.

Patients were selected from the NCR. After notification by the nationwide Dutch PALGA archive, specially trained and on site data managers collected data of all patients on diagnosis, staging, and treatment directly from the patient’s files in all Dutch hospitals.

The following variables were selected for the present study: age, cT-stage, cN-stage, ER status, PR status, HER2 status, histological grade, histological type, multifocality (yes, no or unknown, based on histopathological results of the surgical specimen), use of breast MRI, type of primary surgery (mastectomy or local excision), surgical margin status after local excision (positive or negative), and the use of secondary mastectomy following local excision.

A surgical margin was positive if microscopically confirmed invasive carcinoma, and/or DCIS was present at the inked margin of the surgical specimen. A surgical margin was defined as being more than focally positive if invasive carcinoma or DCIS was present at the inked margin over a length of more than 4 mm, which is also an indication for re-excision or secondary mastectomy [3, 4]. Clinical staging was performed according to the TNM classification (7th edition). Preoperative T-stage was based on the maximum tumor diameter measured on any imaging modality, preferably on breast MRI. Preoperative N-stage was based on clinical and/or radiological examination of the axilla (with tissue sampling of suspect lymph nodes when deemed necessary). The results of a sentinel lymph node biopsy could be classified in the N-stage as this was performed before the start of any treatment. ER and PR receptor status were defined as positive when at least 10% of immunostained nuclei of tumor cells were present. HER2 status was considered positive in case of HER2 3+ (strong and complete membranous expression in >30% of tumor cells) or HER2 2+ (weak complete membranous expression in >10% of tumor cells) confirmed with positive fluorescence in situ hybridization.

All second primary cancers diagnosed in the contralateral breast within three months after the diagnosis of the first breast cancer in the period of 2011–2013 were included and considered as contralateral breast cancers.

### Statistical analysis

The study was divided into an MRI group and a no-MRI group. Differences in patient and disease characteristics between these two groups were tested using χ^2^ test for categorical variables. Multivariable logistic regression analysis was used to determine the association between the use of breast MRI and the following co-variates: year of diagnosis; age at diagnosis; clinical tumor size; clinical nodal status; ER, PR, and HER2 status; tumor grade; histological type; and multifocality. Multivariable logistic regression analysis was also used to test the association between MRI use and the following outcomes: treatment with initial mastectomy (vs. local excision), the presence of more than focally positive surgical margins after local excision, treatment with final mastectomy after previous local excision, and diagnosis of synchronous contralateral breast cancer.

All analyses were performed for the total group of invasive breast cancers and for both ductal and lobular cancers separately. Statistical tests were two-sided, and *p* < 0.05 was considered statistically significant. SAS (version 9.3, SAS Institute, Cary, NC, USA) was used for all statistical analyses.

## Results

For the period of 2011–2013, 36,050 patients were available for the final data analysis. Breast MRI was performed in 10,740 (29.8%) breast cancer patients (Table 1). Almost half of the patients <50 years of age underwent breast MRI: 48.4%, compared to 30.1% of those aged 50–69 years and 15.5% of those aged >70 years. Of the patients with IDC 26.1% underwent breast MRI compared to 54.0% of patients with ILC. Forty-nine percent (49.5%) of the patients in whom multifocal breast cancer was observed underwent breast MRI compared to 26.5% of the patients without multifocal breast cancer. In patients with ILC who underwent breast MRI, tumor stage generally is higher than in patients with IDC (Table 1).

**Table 1:** General patient characteristics

Characteristic	Invasive breast cancer (*n* = 36,050)		Lobular (*n* = 5135)		Ductal (*n* = 28,590)	
	No MRI (%)	MRI (%)	No MRI (%)	MRI (%)	No MRI (%)	MRI (%)
Year of incidence						
2011	8455 (70.4)	3559 (29.6)	842 (48.6)	890 (51.4)	7044 (73.9)	2487 (26.1)
2012	8586 (70.7)	3558 (29.3)	794 (46.1)	928 (53.9)	7159 (74.3)	2472 (25.7)
2013	8269 (69.5)	3623 (30.5)	725 (43.1)	956 (56.9)	6925 (73.5)	2503 (26.5)
Age group (years)						
<50	3495 (51.6)	3282 (48.4)	246 (31.5)	535 (68.5)	3046 (54.1)	2590 (45.9)
50–70	13,974 (69.9)	6023 (30.1)	1168 (41.0)	1683 (59.0)	11,929 (74.6)	4068 (25.4)
70+	7814 (84.5)	1,435 (15.5)	947 (63.0)	556 (37.0)	6153 (88.4)	804 (11.6)
cT stage						
cT1	17,156 (73.4)	6,211 (26.6)	1407 (49.9)	1413 (50.1)	14,663 (76.6)	4489 (23.4)
cT2	6164 (62.9)	3630 (37.1)	657 (38.7)	1042 (61.3)	5036 (67.3)	2443 (32.7)
cT3	377 (43.8)	484 (56.2)	97 (30.6)	220 (69.4)	236 (49.1)	245 (50.9)
cT4	158 (75.2)	52 (24.8)	17 (56.7)	13 (43.3)	128 (78.5)	35 (21.5)
Unknown	1455 (80.0)	363 (20.0)	183 (68.0)	86 (32.0)	1065 (81.0)	250 (19.0)
cN stage						
cN0	22,393 (70.6)	9316 (29.4)	2091 (45.8)	2473 (54.2)	18,692 (74.5)	6383 (25.5)
cN1–3	2575 (66.3)	1312 (33.8)	235 (46.6)	269 (53.4)	2236 (69.1)	1002 (30.9)
Unknown	342 (75.3)	112 (24.7)	35 (52.2)	32 (47.8)	200 (72.2)	77 (27.8)
ER/PR/HER2 status						
ER+ or PR+, and HER2−	18,690 (69.3)	8276 (30.7)	2011 (44.0)	2564 (56.0)	15,468 (74.3)	5356 (25.7)
ER+ or PR+, and HER2+	1829 (67.3)	890 (32.7)	95 (48.2)	102 (51.8)	1660 (68.3)	769 (31.7)
ER− and PR− and HER2−	2692 (74.2)	934 (25.8)	70 (64.2)	39 (35.8)	2356 (74.2)	818 (25.8)
ER− and PR− and HER2+	862 (68.5)	397 (31.5)	12 (41.4)	17 (58.6)	791 (69.3)	350 (30.7)
Unknown	1237 (83.6)	243 (16.4)	173 (76.9)	52 (23.1)	853 (83.5)	169 (16.5)
Grade						
1	6510 (72.5)	2476 (27.6)	368 (42.5)	498 (57.5)	5366 (75.1)	1779 (24.9)
2	10,777 (68.1)	5040 (31.9)	1591 (45.8)	1886 (54.2)	8720 (74.3)	3024 (25.7)
3	7016 (71.7)	2771 (28.3)	256 (50.9)	247 (49.1)	6,351 (72.7)	2385 (27.3)
Unknown	1007 (69.0)	453 (31.0)	146 (50.5)	143 (49.5)	691 (71.6)	274 (28.4)
Multifocality						
No	22,150 (73.5)	8008 (26.5)	1854 (48.6)	1964 (51.4)	18,764 (76.8)	5656 (23.2)
Yes	2738 (50.5)	2688 (49.5)	453 (36.3)	796 (63.7)	2102 (54.2)	1777 (45.8)
Unknown	422 (90.6)	44 (9.4)	54 (79.4)	14 (20.6)	262 (90.0)	29 (10.0)
Margin involvement						
Yes	981 (69.4)	433 (30.6)	156 (53.2)	137 (46.8)	755 (73.8)	268 (26.2)
No	23,474 (69.8)	10,166 (30.2)	2059 (44.3)	2592 (55.7)	19,753 (73.5)	7106 (26.5)
Contralateral breast cancer						
Yes	336 (45.7)	399 (54.3)	52 (32.3)	109 (67.7)	256 (48.7)	270 (51.3)
No	24,974 (70.7)	10,341 (96.3)	2309 (46.4)	2665 (53.6)	20,872 (74.4)	7192 (25.6)

Multivariable analysis (Table 2) showed that patients <50 years of age were more likely to undergo breast MRI compared to patients aged >70 years (OR 6.38, 95% CI 5.89–6.91). Patients with ILC were approximately three times more likely to undergo breast MRI compared to those with IDC (OR 3.48; 95% CI 3.25–3.73). The OR of undergoing breast MRI was 2.35 (95% CI, 2.20–2.50) for patients with multifocal breast cancer compared to those without. Other subgroups which were more likely to undergo breast MRI were those with a clinical tumor size of 2 to 5 cm (cT2) and tumors larger than 5 cm (cT3) or cT4 (when compared to cT1).

**Table 2:** Multivariable analysis of all invasive breast cancer patients (*n* = 36,050) diagnosed in 2011-2013 per defined outcome modality

Outcome characteristic	MRI use			Primary mastectomy			Margin involvement (more than focal)			Secondary mastectomy			Contralateral breast cancer		
	OR	95% CI	*P*	OR	95% CI	*P*	OR	95% CI	*P*	OR	95% CI	*P*	OR	95% CI	*P*
MRI															
No	N/A			1 (Ref)			1 (Ref)			1 (Ref)			1 (Ref)		
Yes				1.22	1.15–1.29	<0001	0.84	0.73–0.97	0.015	1.07	0.89–1.29	0.434	3.55	3.01–4.17	<.0001
Year of incidence															
2011	1 (Ref)			1 (Ref)			1 (Ref)			1 (Ref)			1 (Ref)		
2012	1.02	0.96–1.09	0.468	0.94	0.88–0.99	0.025	0.84	0.73–0.96	0.011	0.79	0.65–0.95	0.012	1.05	0.87–1.26	0.636
2013	1.11	1.04–1.18	0.001	0.93	0.88–0.99	0.020	0.73	0.63–0.84	<.0001	0.80	0.66–0.97	0.026	1.18	0.98–1.41	0.081
Age group (years)															
70+	1 (Ref)			1 (Ref)			1 (Ref)			1 (Ref)			1 (Ref)		
<50	6.38	5.89–6.91	<.0001	0.65	0.60–0.70	<.0001	1.33	1.11–1.60	0.002	1.99	1.56–2.54	<.0001	0.56	0.44–0.71	<.0001
50–69	2.84	2.65–3.04	<.0001	0.46	0.44–0.49	<.0001	0.86	0.74–0.99	0.041	1.02	0.83–1.26	0.848	0.65	0.54–0.77	<.0001
Histology															
Ductal	1 (Ref)			1 (Ref)			1 (Ref)			1 (Ref)			1 (Ref)		
Lobular	3.48	3.25–3.73	<.0001	1.55	1.45–1.67	<.0001	1.94	1.64–2.28	<.0001	2.55	2.06–3.14	<.0001	1.15	0.94–1.39	0.168
Other	0.88	0.78–0.99	0.026	1.01	0.90–1.12	0.920	1.41	1.11–1.80	0.005	1.12	0.79–1.59	0.529	0.95	0.69–1.31	0.754
Clinical tumor size (cT)															
cT1	1 (Ref)			1 (Ref)			1 (Ref)			1 (Ref)			1 (Ref)		
cT2	1.66	1.57–1.76	<.0001	3.17	3.00–3.35	<.0001	1.49	1.29–1.71	<.0001	1.40	1.17–1.69	<.0001	0.57	0.46–0.70	<.0001
cT3–4	2.82	2.44–3.25	<.0001	34.67	26.08–46.09	<.0001	3.21	1.55–6.66	0.002	2.19	0.78–6.16	0.139	0.51	0.30–0.89	0.017
Unknown	0.66	0.57–0.75	<.0001	2.34	2.09–2.62	<.0001	2.42	1.91–3.07	<.0001	2.63	1.93–3.59	<.0001	2.23	1.72–2.88	<.0001
Clinical nodal status (cN)															
cN0	1 (Ref)			1 (Ref)			1 (Ref)			1 (Ref)			1 (Ref)		
cN1–3	0.94	0.87–1.02	0.131	2.53	2.33–2.75	<.0001	1.50	1.21–1.84	0.0001	1.50	1.14–1.98	0.004	0.81	0.59–1.11	0.189
Unknown	1.00	0.78–1.28	0.984	1.42	1.13–1.79	0.003	1.60	0.98–2.64	0.063	1.86	0.97–3.54	0.061	0.82	0.40–1.70	0.599
ER, PR and HER2 status															
ER+ or PR+, and HER2−	1 (Ref)			1 (Ref)			1 (Ref)			1 (Ref)			1 (Ref)		
ER+ or PR+, and HER2+	1.03	0.93–1.13	0.595	1.17	1.07–1.29	0.001	1.30	1.05–1.59	0.014	1.31	1.00–1.74	0.055	0.97	0.69–1.36	0.853
ER− and PR− and HER2−	0.90	0.82–0.99	0.023	1.08	0.99–1.18	0.095	0.71	0.56–0.90	0.004	1.15	0.86–1.54	0.356	0.81	0.55–1.20	0.291
ER− and PR− and HER2+	1.11	0.97–1.27	0.148	1.56	1.36–1.79	<.0001	1.20	0.86–1.66	0.279	1.95	1.30–2.93	0.001	0.56	0.27 –1.14	0.110
Unknown	0.71	0.60–0.85	0.0001	1.18	1.03–1.36	0.021	0.86	0.61–1.22	0.398	1.41	0.91–2.18	0.122	2.33	1.72–3.14	<.0001
Tumor grade															
1	1 (Ref)			1 (Ref)			1 (Ref)			1 (Ref)			1 (Ref)		
2	0.91	0.86–0.97	0.005	1.23	1.15–1.31	<.0001	1.48	1.26–1.73	<.0001	1.59	1.27–1.99	<.0001	0.71	0.60–0.84	<.0001
3	0.79	0.73–0.85	<.0001	1.18	1.09–1.28	<.0001	1.63	1.35–1.97	<.0001	1.87	1.44–2.43	<.0001	0.35	0.26–0.47	<.0001
Unknown	1.06	0.92–1.22	0.409	1.30	1.13–1.48	<.0001	1.98	1.48–2.65	<.0001	1.41	0.93–2.14	0.110	0.86	0.62–1.19	0.354
Multifocality															
No	1 (ref)			1 (Ref)			1 (Ref)			1 (Ref)			1 (Ref)		
Yes	2.35	2.20–2.50	<.0001	4.44	4.15–4.76	<.0001	2.85	2.44–3.34	<.0001	4.78	3.92–5.82	<.0001	1.02	0.84–1.25	0.831
Unknown	0.39	0.27–0.55	<.0001	1.48	1.16–1.90	0.002	3.46	1.63–7.35	0.001	3.89	1.42–10.6	0.008	0.87	0.45–1.71	0.688
Margin involvement															
No										1 (Ref)					
Yes	N/A	N/A	N/A	N/A	N/A	N/A	N/A	N/A	N/A	41.94	35.68–49.30	<0.001	N/A	N/A	N/A

### Primary mastectomies

Table 3 shows that patients with IDC that underwent breast MRI had a higher likelihood of being treated with primary mastectomy than those without (OR 1.30, 95% CI 1.22–1.39). Patients with ILC who underwent preoperative breast MRI had a slightly decreased likelihood of being treated with primary mastectomy than those without a breast MRI exam (OR 0.86, 95% CI 0.76–0.99, Table 4). Table 2 shows that the likelihood of primary mastectomy was higher for ILC patients when compared to those with IDC (OR 1.55, 95% CI 1.45–1.67 versus OR 1.01, 95% CI 0.90–1.12, respectively).

**Table 3:** Multivariable analysis of all patients with invasive ductal breast cancer (*n* = 28,590) in the period 2011–2013 per defined outcome modality

Outcome characteristic	MRI use			Primary mastectomy			Margin involvement (more than focal)			Secondary mastectomy			Contralateral breast cancer		
	OR	95% CI	*P*	OR	95% CI	*P*	OR	95% CI	*P*	OR	95% CI	*P*	OR	95% CI	*P*
MRI															
No				1 (Ref)			1 (Ref)						1 (Ref)		
Yes	N/A			1.30	1.22–1.39	<.0001	0.90	0.77–1.06	0.202	1.23	1.00–1.53	0.054	4.07	3.38–4.90	<.0001
Year of incidence															
2011	1 (Ref)			1 (Ref)			1 (Ref)						1 (Ref)		
2012	1.02	0.95–1.09	0.629	0.93	0.87–0.99	0.025	0.83	0.71–0.98	0.025	0.84	0.67–1.05	0.122	0.95	0.76–1.18	0.635
2013	1.08	1.01–1.16	0.029	0.93	0.87–1.00	0.047	0.75	0.64–0.89	0.001	0.90	0.72–1.13	0.363	1.14	0.92–1.40	0.242
Age group (years)															
70+	1 (Ref)			1 (Ref)			1 (Ref)						1 (Ref)		
<50	6.89	6.27–7.56	<.0001	0.63	0.58–0.68	<.0001	1.29	1.05–1.59	0.014	1.94	1.45–2.58	<.0001	0.47	0.35–0.63	<.0001
50−69	2.94	2.70–3.19	<.0001	0.46	0.43–0.49	<.0001	0.82	0.69–0.98	0.027	0.97	0.75–1.25	0.819	0.64	0.52–0.79	<.0001
Clinical tumor size (cT)															
cT1	1 (Ref)			1 (Ref)			1 (Ref)						1 (Ref)		
cT2	1.66	1.56–1.77	<.0001	3.10	2.87–3.25	<.0001	1.42	1.21–1.68	<.0001	1.29	1.04–1.61	0.023	0.63	0.49–0.81	<.001
cT3–4	3.08	2.57–3.69	<.0001	36.35	25.30–52.23	<.0001	3.61	1.36–9.60	0.010	0.45	0.07–2.98	0.409	0.57	0.26–1.22	0.147
Unknown	0.71	0.61–0.82	<.0001	2.38	2.09–2.69	<.0001	2.59	1.97–3.40	<.0001	2.92	2.04–4.20	<.0001	2.05	1.49–2.82	<.0001
Clinical nodal status (cN)															
cN0	1 (Ref)			1 (Ref)			1 (Ref)						1 (Ref)		
cN1–3	0.96	0.88–1.05	0.410	2.47	2.26–2.69	<.0001	1.49	1.19–1.86	0.001	1.59	1.18–2.14	0.003	0.82	0.61–1.23	0.423
Unknown	1.02	0.77–1.36	0.880	1.42	1.09–1.86	0.010	1.46	0.79–2.70	0.223	1.43	0.62–3.28	0.400	0.87	0.33–2.03	0.667
ER, PR and HER2 status															
ER+ or PR+, and HER2−	1 (Ref)			1 (Ref)			1 (Ref)						1 (Ref)		
ER+ or PR+, and HER2+	1.08	0.97–1.19	0.155	1.15	1.04–1.27	0.007	1.36	1.09–1.69	0.006	1.27	0.94–1.71	0.12	0.95	0.66–1.38	0.801
ER− and PR− and HER2−	0.93	0.84–1.03	0.156	1.09	0.99–1.20	0.077	0.72	0.56–0.92	0.009	1.08	0.78–1.49	0.649	0.84	0.56–1.28	0.421
ER− and PR−and HER2+	1.10	0.96–1.27	0.183	1.60	1.39–1.85	<.0001	1.18	0.83–1.67	0.351	2.13	1.40–3.25	0.001	0.45	0.20–1.03	0.057
Unknown	0.88	0.72–1.07	0.195	1.11	0.94–1.31	0.206	0.84	0.56–1.27	0.413	1.52	0.91–2.56	0.114	2.31	1.61–3.32	<.0001
Tumor grade															
1	1 (Ref)			1 (Ref)			1 (Ref)						1 (Ref)		
2	0.91	0.84–0.97	0.008	1.21	1.13–1.30	<.0001	1.62	1.34–1.95	<.0001	1.91	1.44–2.52	<.0001	0.72	0.59–0.88	0.001
3	0.78	0.71–0.85	<.0001	1.17	1.07–1.27	<.001	1.76	1.42–2.18	<.0001	2.21	1.63–3.01	<.0001	0.35	0.26–0.48	<.0001
Unknown	1.09	0.93–1.28	0.306	1.43	1.22–1.68	<.0001	2.57	1.82–3.62	<.0001	1.72	1.02–2.90	0.043	0.82	0.54–1.24	0.341
Multifocality															
No	1 (ref)			1 (Ref)			1 (Ref)						1 (Ref)		
Yes	2.62	2.43–2.82	<.0001	4.70	4.34–5.09	<.0001	2.88	2.39–3.47	<.0001	4.41	3.47–5.60	<.0001	0.82	0.63–1.06	0.129
Unknown	0.38	0.25–0.57	<.0001	1.77	1.33–2.34	<.0001	3.15	1.18–8.45	0.023	1.72	0.40–7.37	0.466	0.82	0.37–1.84	0.634
Margin involvement															
No															
Yes	N/A	N/A	N/A	N/A	N/A	N/A	N/A	N/A	N/A	45.51	37.69–54.96	<0.0001	N/A	N/A	N/A

**Table 4:** Multivariable analysis of all patients with invasive lobular breast cancer (*n* = 5135) in the period 2011–2013

Outcome characteristic	MRI use			Primary mastectomy			Margin involvement (more than focal)			Secondary mastectomy			Contralateral breast cancer		
	OR	95% CI	*P*	OR	95% CI	*P*	OR	95% CI	*P*	OR	95% CI	*P*	OR	95% CI	*P*
MRI															
No	N/A	N/A	N/A	1 (Ref)			1 (Ref)						1 (Ref)		
Yes				0.86	0.76–0.99	0.030	0.59	0.44–0.79	<.001	0.61	0.61	0.009	2.50	1.73–3.61	<.0001
Year of incidence															
2011	1 (Ref)			1 (Ref)			1 (Ref)						1 (Ref)		
2012	1.14	0.99**–**1.32	0.061	1.04	0.89–1.22	0.598	0.93	0.67**–**1.27	0.632	0.67	0.45–1.02	0.059	1.44	0.96**–**2.16	0.079
2013	1.30	1.13**–**1.51	<.001	0.98	0.84**–**1.15	0.814	0.61	0.43**–**0.87	0.006	0.57	0.37**–**0.89	0.012	1.43	0.95**–**2.16	0.090
Age group (years)															
70+	1 (Ref)			1 (Ref)			1 (Ref)						1 (Ref)		
<50	4.01	3.31**–**4.87	<.0001	0.70	0.57**–**0.86	0.001	1.38	0.87**–**2.19	0.173	2.14	1.20–**3.82**	0.010	0.85	0.52**–**1.38	0.498
50−69	2.71	2.37**–**3.10	<.0001	0.50	0.43**–**0.58	<.0001	1.09	0.77**–**1.54	0.640	1.22	0.78**–**1.92	0.378	0.65	0.44**–**0.95	0.025
Clinical tumor size (cT)															
cT1	1 (Ref)			1 (Ref)			1 (Ref)						1 (Ref)		
cT2	1.83	1.60**–**2.09	<.0001	3.95	3.43**–**4.55	<.0001	1.74	1.26**–**2.40	0.001	2.17	1.44**–**3.25	<.001	0.35	0.220.56	<.0001
cT3–4	2.69	2.08**–**3.47	<.0001	31.59	19.12**–**52.2	<.0001	2.95	0.88**–**9.87	0.079	7.28	1.85–28.62	0.005	0.45	0.19**–**1.04	0.063
Unknown	0.53	0.39**–**0.71	<.0001	2.23	1.67**–**2.99	<.0001	2.08	1.14**–**3.80	0.020	1.86	0.89**–**3.91	0.100	3.22	1.95**–**5.33	<.0001
Clinical nodal status (cN)															
cN0	1 (Ref)			1 (Ref)			1 (Ref)						1 (Ref)		
cN1–3	0.77	0.63**–**0.94	0.001	2.82	2.18**–**3.65	<.0001	1.15	0.60**–**2.19	0.670	1.31	0.61**–**2.79	0.493	0.76	0.38**–**1.53	0.439
Unknown	0.96	0.57**–**1.61	0.866	1.58	0.92**–**2.73	0.099	2.24	0.83**–**6.01	0.110	4.07	1.25–13.32	0.020	0.85	0.25**–**2.86	0.792
ER, PR and HER2 status															
ER+ or PR+, and HER2−	1 (Ref)			1 (Ref)			1 (Ref)						1 (Ref)		
ER+ or PR+, and HER2+	0.75	0.56**–**1.02	0.067	1.49	1.06**–**2.09	0.020	0.67	0.28**–**1.61	0.372	0.87	0.29**–**2.60	0.804	1.31	0.56**–**3.05	0.536
ER− and PR− and HER2−	0.52	0.34**–**0.79	0.002	1.19	0.74**–**1.92	0.476	1.17	0.43**–**3.18	0.762	2.50	0.77**–**8.13	0.127	0.42	0.06**–**3.09	0.395
ER− and PR− and HER2+	1.35	0.62**–**2.98	0.451	1.66	0.71**–**3.92	0.245	0.70	0.08**–**6.06	0.748	1.51	0.15**–**15.3	0.727	4.02	0.79**–**20.35	0.093
Unknown	0.36	0.25**–**0.53	<.0001	1.70	1.18**–**2.44	0.004	0.49	0.18**–**1.39	0.183	0.60	0.17**–**2.07	0.414	1.98	1.01**–**3.90	0.047
Tumor grade															
1	1 (Ref)			1 (Ref)			1 (Ref)						1 (Ref)		
2	0.81	0.69**–**0.95	0.011	1.33	1.12**–**1.57	0.001	0.96	0.68**–**1.37	0.833	0.94	0.61**–**1.47	0.794	0.70	0.48**–**1.04	0.077
3	0.60	0.47**–**0.76	<.0001	1.39	1.06**–**1.81	0.016	1.55	0.90**–**2.65	0.113	1.11	0.56**–**2.22	0.763	0.36	0.15**–**0.87	0.024
Unknown	0.97	0.72**–**1.32	0.860	1.15	0.83**–**1.58	0.400	0.99	0.50**–**1.94	0.964	0.66	0.27**–**1.59	0.351	0.83	0.43**–**1.63	0.597
Multifocality															
No	1 (ref)			1 (Ref)			1 (Ref)						1 (Ref)		
Yes	1.59	1.38–1.82	<.0001	3.81	3.26**–**4.45	<.0001	2.39	1.71–3.34	<.0001	6.74	4.50–10.08	<.0001	1.48	1.04**–**2.09	0.029
Unknown	0.40	0.21**–**0.76	0.006	0.66	0.37**–**1.18	0.162	5.00	1.42–17.53	0.012	13.79	2.94–64.6	0.001	1.29	0.36**–**4.56	0.695
Margin involvement															
No										1 (Ref)					
Yes	N/A	N/A	N/A	N/A	N/A	N/A	N/A	N/A	N/A	34.77	23.84–50.71	<0.0001	N/A	N/A	N/A

### Positive surgical margins and secondary mastectomies

The association between breast MRI use and the lower risk of positive surgical margins was not statistically significant for patients with IDC (OR 0.90, 95% CI 0.77–1.06, Table 3). It was strongest in patients with ILC (OR 0.59, 95% CI 0.44–0.79, Table 4). In absolute percentages, 3.6% of the IDC patients that underwent breast MRI had positive surgical margins, versus 3.7% in those who had not. For ILC patients, the use of breast MRI resulted in a difference between positive surgical margins of 5.0% for those with breast MRI versus 7.0% for those without.

Regarding the association of breast MRI and secondary mastectomy, the subgroup of patients with IDC did not show a statistically significant lower risk of secondary mastectomy when breast MRI was used (OR 1.23, 95% CI 1.00–1.53, Table 3). In contrast, in patients with ILC to likelihood of a secondary mastectomy, it was significantly lower when breast MRI was used (OR 0.61, 95% CI 0.42–0.88, Table 4).

In young patients, breast MRI was associated with less primary mastectomies but with an increase in surgical margin involvement (OR 1.33, 95% CI 1.11–1.60) and an increase in secondary mastectomy rates (OR 1.99, 95% CI 1.56–2.54, Table 2).

Irrespective of breast MRI use, it remains important to realize that the likelihood of having positive surgical margins was almost two times higher in patients with ILC when compared to patients with IDC (OR 1.94, 95% CI 1.64–2.28, Table 2). Patients with multifocal breast cancer were three times more likely to have positive surgical margins when compared to non-multifocal breast cancers (OR 2.85, 95% CI 2.44–3.34, Table 2). Furthermore, the risk of having positive surgical margins was higher in young patients, larger tumors, and an intermediate or poor tumor grade (Table 2).

### Contralateral tumors in invasive breast cancers

Patients who underwent breast MRI were almost four times more frequently diagnosed with contralateral breast cancer (Table 2), compared to those in whom breast MRI was not performed (3.9% vs. 1.3% cases, respectively: OR 3.55, 95% CI 3.01–4.17). Contralateral breast cancer was less frequently observed in women <70 years when compared to women >70 years, in those with increasing tumor size and less favorable histologic grade. No significant difference in the impact of breast MRI on the risk of contralateral breast cancer could be observed between primary IDC or ILC.

## Discussion

In our current study population of 36,050 Dutch patients, we found that breast MRI was performed in 29.8% of all patients. For most patients (those with IDC), the use of breast MRI increases the number of mastectomies without any improvement in surgical outcome. In contrast, we observed that breast MRI use was associated with a lower risk of primary and secondary mastectomies in patients with ILC. Although breast MRI was more often used in larger tumors, size did not seem to affect our observations, as in every sub group still a significant number of the smallest (i.e., cT1), tumors were present and the multivariable analysis takes these variations into account. We also found that patients undergoing breast MRI were four times more frequently diagnosed with contralateral breast cancers.

Our results are in line with previous results from a large meta-analysis covering nine eligible studies (3112 patients) [5]. In this study, patients were more likely to undergo mastectomy when they underwent breast MRI: adjusted OR 3.06 (95% CI 2.03–4.62, *p* < 0.001). However, re-excision rates were comparable for both study groups: adjusted OR 0.95 (95% CI 0.73–1.24, *p* = 0.71). In our study, mastectomy rates were also increased for patients that underwent breast MRI (OR 1.22), with only a slight improvement in surgical margin involvement (OR 0.84), mainly attributable to patients with ILC. The fact that breast MRI is not able to improve surgical margin involvement is poorly understood. In a recent publication, it was suggested that we might be performing breast MRI in the wrong position [8]. Tumor metrics, such as changes in volume, surface area, compactness, sphericity, and distances from key landmarks varied from 6.5 to 23.8% when standard (prone) breast MRI was compared with (supine) intraoperative breast MRI. In addition, the authors could directly assess any residual tumor tissue during surgery. These novel insights should be studied in a larger study but appear promising in achieving improved surgical outcomes when using breast MRI, maybe even regardless of tumor type.

ILCs are difficult to detect on conventional imaging [9, 10]. Sensitivity of digital mammography for detecting ILC varies from 57 to 79%, and sensitivity is only slightly better when ultrasound is used: 81–83%. ILC is reported to be associated with higher rates of surgical margin involvement than any other invasive breast cancer subtype, with reported re-excision rates varying widely from 39 to 80% [11–15]. However, these studies have not taken preoperative breast MRI into account, which is the most accurate imaging modality to evaluate the extent of ILC. To our knowledge, only four studies evaluated the impact of preoperative breast MRI in patients with ILC on surgical outcomes [5].

Mann et al. were the first to demonstrate that preoperative breast MRI in ILC could reduce re-excision rates (9 vs. 27% in the group not receiving breast MRI) without increasing the rate of mastectomies (48 vs. 59%, *p* = 0.098) [16]. Conflicting results were subsequently published by McGhan et al. who did not observe any significant differences in re-excision rates in their single-center study: 9.2% for the non-MRI group versus 4.2% for the MRI group in re-excision of margins only (*p* = 0.202) [17]. Although their results were not statistically significant, they nevertheless showed a similar trend as the results observed by Mann et al. and our current findings. Conversion to mastectomy after primary BCT did not differ statistically significant between study groups: 7.3% for the non-MRI group versus 2.8% for the MRI group (*p* = 0.189). In another single-center study, the number of primary mastectomies between the MRI and the non-MRI groups was 38% versus 30%, respectively (*p* = 0.119), and the number of re-excision with wider margins did not differ between groups either: 11% for the MRI group and 9% for the non-MRI group (*p* = 0.322) [18]. The COMICE trial by Turnbull et al. was a prospective randomized controlled trial evaluating the use of preoperative breast MRI [19]. In this study, 1623 subjects were randomized between preoperative breast MRI or not. In patients with ILC (*n* = 133), rates of primary mastectomies appeared to be higher in those who had breast MRI as compared to those who did not (8.8 vs. 2.8%), with no clear difference between secondary mastectomy rates (19.4 vs. 15.7%).

Since ILCs only compromise 10–15% of all invasive breast cancers, the before mentioned studies all included a limited number of ILC cases, hampering us to draw definite conclusions. Our current study, although retrospective, has the advantage of having a much larger sample size of patients with ILC (*n* = 5135) and summarizes the results in a population-based analysis, which considers the variation in expertise that exists between breast cancer teams and hospitals, including general, teaching, and university hospitals. We demonstrated that preoperative evaluation using breast MRI of patients with ILC and treated with primary surgery is beneficial, most likely to the superior ability of breast MRI to delineate tumor extent [1]. Its use in ILC patients reduced the number of (primary and secondary) mastectomies and positive surgical margins. The results of our study show that prior European recommendations on the use of breast MRI in patients with ILC [20, 21] can indeed be translated to everyday clinical practice and different hospital subtypes. Nevertheless, one should realize that irrespective of breast MRI use, the likelihood of having positive surgical margins remains almost two times higher in patients with ILC when compared to patients with IDC (OR 1.94, 95% CI 1.64–2.28, Table 2) and patients with ILC undergo mastectomy more often than IDC patients: 31.0 versus 12.1%, respectively (Table 1).

Even though breast MRI does not seem beneficial for surgical outcomes in especially IDC patients, it might still be considered for evaluation of the contralateral breast as the likelihood of detecting contralateral breast cancer was almost fourfold higher when compared with patients that did not undergo breast MRI. Lehman et al. detected non-symptomatic, contralateral breast cancers using breast MRI in 3.1% of the cases [22], with a wide range of 4–24% reported in previous studies [23]. Previously, Houssami et al. evaluated the impact of contralateral breast cancer detection and reported a relative increase in survival due to its early detection. However, these contralateral breast cancers were detected in any kind of way [24]. In addition, studies on adjuvant systemic therapy have consistently shown to reduce the risk of contralateral breast cancer during follow-up, suggesting that already at the diagnosis of the primary breast cancer a secondary, contralateral breast cancer may be present [25, 26]. Whether knowledge of the presence of a contralateral tumor solely detected by breast MRI and its treatment ultimately improves breast cancer outcome formally remains to be proven. In our study, patients that underwent breast MRI had an almost fourfold increased risk of having contralateral breast cancer compared to those in whom breast MRI was not performed (3.9 vs. 1.3%, respectively, Table 1). From our data, contralateral breast cancers seem to be more frequently detected by breast MRI in elderly patients and patients with smaller tumor sizes, which is in line with results from the Netherlands of an older research period (1989–2009) [27]. In this latter study, it was pointed out that routine use of breast MRI in older patients is questionable, since its added value in a patient group with presumably more comorbidities might be limited. However, we might also presume that patient selection in this population-based analysis may already have accounted for performance score. Because older patients may have been treated less often with adjuvant chemotherapy, one may also argue that especially these patients may benefit from breast MRI to prevent the development of symptomatic contralateral breast cancer. Future studies should investigate the prognostic impact of these findings and the cost-effectiveness of preoperative breast MRI for detecting occult contralateral breast cancers in the elderly, perhaps in comparison to other novel imaging modalities, such as (automated) breast ultrasound [28], digital breast tomosynthesis [29], or contrast-enhanced spectral mammography [30].

The current study design resulted in several limitations. First, in this retrospective analysis, several parameters (such as breast size and density, tumor localization within the breast, patient breast cancer risk profile, and the initial surgical treatment plan based on mammographic and/or ultrasound findings) were not available since this database was primarily designed for monitoring quality of delivered health care. Hence, there is a risk of residual confounding since the motivation for performing the breast MRI exam cannot be extracted from this database. Thus, we do not have a clear idea of factors that prompted MRI. We cannot exclude that in IDC cases, the multidisciplinary tumor board requested MRI as a confirmation for mastectomies based on either tumor size or suspected multicentricity at conventional imaging. On the other hand, we cannot exclude that for ILC cases the board had more propensity to ask for breast MRI to avoid mastectomy. In short, it is important to know that ‘association is not causation.’ However, the MIPA study sheds more light on breast MRI indications [31]. As in daily practice, breast MRI is mostly used in case of doubt on feasibility of breast-conserving surgery. Therefore, we feel confident in concluding that breast MRI use in patients with ILC (in contrast to patients with IDC) protects against an overuse of mastectomy. Second, survival outcome is not (yet) available for this population, which is the most important clinical outcome in this setting of course. Although an individual patient data meta-analysis showed that there was no difference in 8-year local recurrence-free survival between breast cancer patients undergoing breast MRI and those who did not, most cases consisted of patients with IDC with only 6–8% of the cases in each study group consisting of ILC patients. Thus, to the best of our knowledge, there is currently no sound evidence on the impact of preoperative breast MRI on survival outcomes specifically for patients with ILC [32].

In conclusion, breast MRI use was associated with a reduced number of mastectomies and less positive surgical margins in invasive lobular cancer, but with an increased number of mastectomies in ductal cancers. Breast MRI use was associated with a fourfold higher incidence of CBC.

## Abbreviations

BCT: Breast conservative therapy
CI: Confidence interval
DCIS: Ductal carcinoma in situ
ER: Estrogen receptor
EUSOBI: European Society of Breast Imaging
HER2: HER2 receptor
IDC: Invasive ductal cancer
ILC: Invasive lobular cancer
MIPA: Multicenter International Prospective Meta-analysis (study)
MRI: Magnetic resonance imaging
NCR: Netherlands Cancer Registry
OR: Odds ratio
PR: Progesterone receptor
PALGA: Dutch Pathology Archives of histo- and cytopathology
